# A nationwide evaluation study of the quality of care and respect of human rights in mental health facilities in Ghana: results from the World Health Organization QualityRights initiative

**DOI:** 10.1186/s12889-022-13102-2

**Published:** 2022-04-02

**Authors:** Maria Francesca Moro, Mauro Giovanni Carta, Leveana Gyimah, Martin Orrell, Caroline Amissah, Florence Baingana, Humphrey Kofie, Dan Taylor, Nurokinan Chimbar, Martha Coffie, Celline Cole, Joana Ansong, Sally-ann Ohene, Priscilla Elikplim Tawiah, Michela Atzeni, Silvia D’Oca, Oye Gureje, Michelle Funk, Nathalie Drew, Akwasi Osei

**Affiliations:** 1 Columbia University Irving Medical Center, New York, US USA; 2 University of Cagliari, Cagliari, IT Italy; 3 WHO Country Office for Ghana, Accra, GH Ghana; 4 Institute of Mental Health, University of Nottingham, Nottingham, UK; 5 Ghana Ministry of Health - Mental Health Authority, Accra, GH Ghana; 6 WHO Regional Office for Africa, Brazzaville, CG Congo; 7 Mental Health Society of Ghana, Accra, GH Ghana; 8 MindFreedom Ghana, Accra, GH Ghana; 9 Methods Consult, Accra, GH Ghana; 10 Charité University Medicine Berlin, Berlin, DE Germany; 11 Department of Psychiatry, WHO Collaborating Centre for Research and Training in Mental Health, Neurosciences and Substance Abuse, University of Ibadan, Ibadan, NG Nigeria; 12 Policy, Law and Human Rights, Department of Mental Health & Substance Use, World Health Organization, Geneva, CH Switzerland

**Keywords:** WHO QualityRights, Human Rights, Psychiatric services, Ghana, UN CRPD, Mental healthcare, Quality of care

## Abstract

**Background:**

In 2012, Ghana ratified the United Nations Convention on the Rights of Persons with Disabilities and enacted a Mental Health Act to improve the quality of mental health care and stop human rights violations against people with mental health conditions. In line with these objectives, Ghanaian stakeholders collected data on the quality of mental health services and respect for human rights in psychiatric facilities to identify challenges and gather useful information for the development of plans aimed to improve the quality of the services offered. This study aimed to assess psychiatric facilities from different Ghanaian regions and provide evidence on the quality of care and respect of human rights in mental health services.

**Methods:**

Assessments were conducted by independent visiting committees that collected data through observation, review of documentation, and interviews with service users, staff, and carers, and provided scores using the World Health Organization QualityRights Toolkit methodology.

**Results:**

This study revealed significant key challenges in the implementation of the United Nations Convention on the Rights of Persons with Disabilities principles in Ghanaian psychiatric services. The rights to an adequate standard of living and enjoyment of the highest attainable standard of health were not fully promoted. Only initial steps had been taken to guarantee the right to exercise legal capacity and the right to personal liberty and security. Significant gaps in the promotion of the right to live independently and be included in the community were identified.

**Conclusions:**

This study identifies shortcomings and critical areas that the Ghanaian government and facilities need to target for implementing a human rights-based approach in mental health and improve the quality of mental health care throughout the country.

## Background

The World Health Organization (WHO) estimates that, out of 28 million Ghanaians, around 2.3 million people live with a mental health condition and are in need of mental health care. However, only 2% of them receive treatment and support in psychiatric services [1, 2]. One of the reasons why this happens is that mental health services in Ghana are significantly underfunded. Ghana's health expenditure is roughly 4.5% of the gross national product [3], with only 1.3% of the health expenditure allocated to mental health [4]. Most of the mental health budget (80%) goes to the maintenance of the three government psychiatric hospitals, Accra, Pantang, and Ankaful, although psychiatric in-patient units are present also in 5 of the 10 regional general hospitals in the country [5]. The ratio of psychiatric beds in mental health facilities in or around the capital, Accra, to the total number of beds in the rest of the country is around 6.28 to 1 [5]. In addition, most mental health professionals (psychiatrists, psychiatric nurses, medical doctors, psychologists, social workers, and occupational therapists) work in mental health facilities located in the main cities of the country, indicating that the allocation of resources for mental health is very much skewed towards urban areas [5, 6]. Community mental health care exists in Ghana, but it is not well developed and does not cover the whole country [3]. Due to the difficulty in accessing mental health services and the widespread beliefs about the supernatural causation of mental ill health, most Ghanaians with a mental health condition, especially in rural areas, receive needed care from faith-based and traditional healers [7, 8].

In addition to facing challenges in providing access to mental health services, Ghana has recently come under scrutiny for human rights violations against people with mental health conditions, as reflected in several reports by local and international organizations [9–11]. According to these reports, Ghanaians with mental health conditions in psychiatric facilities are exposed to poor conditions in regard to physical infrastructure, food inadequacy, and overcrowding. Many residents are forced to live in these institutions against their will and without any possibility to challenge their situation. Incidents of verbal and physical abuses have been reported against service users who try to escape and fail to take medication or follow hospital rules. There are also reports suggesting that unmodified electroconvulsive therapy (i.e., without anesthesia) is used and people are sometimes isolated in seclusion rooms for up to three days, although such practices constitute ill-treatment according to the United Nations Special Rapporteur on Torture.

In 2012, the government of Ghana ratified the United Nations Convention on the Rights of Persons with Disabilities (UN CRPD) and enacted a Mental Health Act to improve the quality of mental health care (including the provision of better access to services) and eliminate the human rights violations against people with mental health conditions in psychiatric facilities and the community [12]. The Act created a Mental Health Authority within the Ministry of Health, for which the initial priorities were the improvement of the quality of mental health care and the promotion of the rights of Ghanaians with mental health conditions. In February 2019, the Mental Health Authority and non-governmental organizations including organizations of people with mental health conditions launched the “QualityRights in Mental Health, Ghana” initiative. This project aims to transform mental health services and promote the rights of persons with mental health conditions in Ghana [13]. As part of this project, Ghanaian stakeholders collected data on the quality of mental health care and respect of human rights in psychiatric facilities in their country, to identify the key challenges and gather useful information for the development of plans aimed to improve the quality of the services offered. These stakeholders chose to collect data using the WHO QualityRights Assessment Toolkit [14], an instrument developed to evaluate the quality of care and respect of human rights in mental health facilities and designed for global applicability. The WHO QualityRights Assessment Toolkit follows the recent human rights framework of the United Nations Convention on the Rights of Persons with Disabilities (UN CRPD) [15] and has been previously used in other countries, such as India [16], Chile [17], Czech Republic [18], and Tunisia [19]. In 2018, this instrument was also used for evaluating four mental health facilities in West Africa, including Pantang Psychiatric Hospital in Ghana [20].

The present study builds on this previous work and, in line with the efforts of the Ghana government to tackle human rights violations in mental health, aims to evaluate psychiatric facilities from different Ghanaian regions and provide evidence on the quality of care and respect of human rights in mental health services throughout the country.

## Methods

### Aim and design of the study

This is a mixed-methods convergent design study aimed to assess psychiatric facilities from different Ghanaian regions and provide evidence on the quality of care and respect of human rights in mental health services.

### Setting of the study

We conducted the study in seven different mental health facilities: Accra Psychiatric Hospital, Ankaful Psychiatric Hospital, Eastern Regional Hospital, Ho Teaching Hospital, Komfo Anokye Hospital, Sunyani Regional Hospital, and Korle Bu Teaching Hospital (psychiatric facility). These mental health facilities are located in different Ghanaian regions and were selected to provide a representative picture of the quality of care and respect of human rights in psychiatric services throughout the country.

### Evaluation visits

In each facility, the assessment was conducted by independent visiting committees. Members of the visiting committees were selected from multidisciplinary backgrounds and also included 1 Human rights advocate from NGO, 2 service users, 5 Mental health advocates from NGO, 1 Psychiatrist, 2 Intellectual Disability Organization advocates, 1 Psychosocial Disability Organization advocate and 1 Researcher. A three-day training was organised in Accra for 34 participants (10 women, 24 men) to build their capacity on the evaluation of the quality of care and human rights respect in mental health facilities (using the WHO Quality Rights Toolkit). Thirteen visiting committees’ members were selected from the pool of participants based on their availability and personal background (to guarantee a broad range of expertise and skills). At least one service user was present in all the visits to the facilities. To ensure the committee was well-trained on the methodology for the assessment, there was a simulation exercise at the Pantang Psychiatric Hospital in Accra. The evaluation visits were planned in collaboration with the management staff in each facility. During the evaluation visits, the visiting committees carried out an observation of the facilities, the review of the documentation, and interviews with service users, their carers, and staff members. Visiting committee members collected both quantitative (e.g., data on length of stay at the hospital, number of users per ward, quantity of essential psychotropic medications) and qualitative data (e.g., photographs of the facilities’ physical environment, notes regarding the presence of policies and guidelines, notes regarding the information in medical files and nurses’ charts, notes based on the interviews including verbatim quotes by respondents). All the visiting committees’ members collected data through observation. The retired psychiatrist was assigned to carry out the documentation review because of his wealth of experience in direct mental health practice. The researcher was designated as rapporteur to collate notes and compile results while the rest of the trained visiting committee members collected data through interviews. In between the assessments, the coordinating team continued to organise virtual meetings through zoom to discuss challenges and feedback from the field for redress. Assessment reports were written for each of the facilities evaluated, with recommendations for improving the quality of care and respect of human rights.

### Instrument

**Fig. 1:**
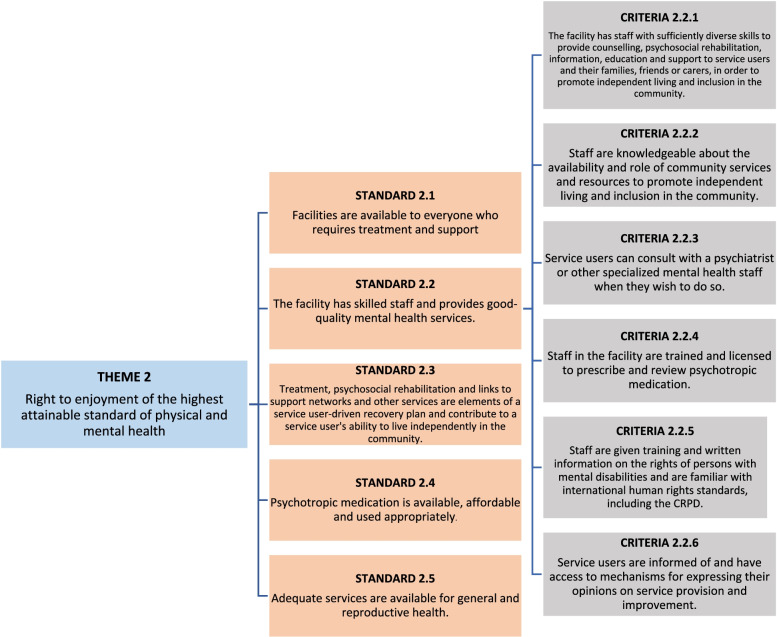
Example of the WHO QualityRights Toolkit’s organization, with the division of themes into standards and criteria

The quality of care and respect of human rights in mental health facilities were assessed using the WHO QualityRights Toolkit [14]. This instrument is based on the modern human rights framework of the United Nations Convention on the Rights of Persons with Disabilities and includes five themes. Each theme focuses on a specific UN CRPD right: **1)** The right to an adequate standard of living (Article 28); **2)** The right to enjoyment of the highest attainable standard of physical and mental health (Article 25), **3)** The right to exercise legal capacity and the right to personal liberty and security of person (Articles 12 and 14); **4)** Freedom from torture or cruel, inhuman or degrading treatment or punishment and from exploitation, violence and abuse (Articles 15 and 16); and **5)** The right to live independently and be included in the community (Article 19). The themes are organized into standards, which consist of different criteria (see the example in Fig. 1). The WHO QualityRights Toolkit has been previously used in several countries [16–19], including Ghana [20]. Although the Toolkit has not been formally validated for use in Ghana, the members of the visiting committees checked its contents and adapted them to the local context (e.g., adding prompts relevant to Ghana in the interview instrument). The adapted WHO QualityRights Toolkit was pilot tested before the evaluations started, during a training simulation exercise at the Pantang Psychiatric Hospital in Accra.

### Analyses

The visiting committee members integrated qualitative and quantitative data using a mixed methods convergent design [21, 22]. First, in all the facilities assessed, they assigned a score to each theme, standard, and criterion using a mixed-method approach, based on 1) the data collected with the observation of the facility, the review of the documentation, and the interviews with service users, their carers, and staff and 2) extensive discussions among visiting committee members. During the discussions, the rapporteur presented the notes and photographs from the visits and the visiting committee members integrated with their own notes when needed. Each theme, standard and criterion was scored as follows: “Not initiated—N/I,” “Achievement initiated—A/I,” “Achieved partially—A/P” “Achieved in full—A/F”, or “Not applicable – N/A.” First, the visiting committee members evaluated criteria. Then, based on the scores at the criteria, a score was assigned to the corresponding standard. Finally, scores at the standards were used to assign a score at each theme. Using the ratings, provided by the visiting committees, for each standard, we calculated the total number of criteria (with corresponding percentages) that received the different scores (i.e., N/I, A/I, A/P, A/F, N/A) in all the facilities evaluated. These percentages were then presented in the form of bar charts reflecting the adherence to the rights represented by each theme. Finally, we analyzed the qualitative descriptions and justifications provided by the visiting committees in the reports to identify areas for improvement surrounding each of the five themes and provide recommendations. The same analytical method has been used for analyzing the data from evaluations carried out in other countries.

## Results

### Details on the assessment team’s visits

During each visit, the assessment team conducted an observation of the facility, a review of the documentation, and interviews with service users, their family members, friends, or caregivers, and staff members. Table 1 provides information on the number of staff members and service users in each facility, the number of interviews completed, and the length of the visits.

**Table 1:** Profile of the mental health facilities evaluated, and details of the assessment team’s visits

**Mental health** **facility**	**N. of** **Staff members**^a^	**No. of Service users**^b^	**Staff** **members** **Interviewed**	**Service Users** **Interviewed**	**Carers (family member/friend) Interviewed**	**Length of the visit**
Accra Psychiatric Hospital	782	319	57	68	34	2 days
Ankaful Psychiatric Hospital	462	236	34	53	26	2 days
Eastern Regional Hospital	15	20	12	19	9	2 days
Ho Teaching Hospital	37	10	7	19	9	3 days
Komfo Anokye Hospital	73	80	7	17	8	2 days
Sunyani Regional Hospital	19	22	7	14	7	2 days
Korle Bu Teaching Hospital	74	32	7	15	6	2 days

^a^Data on in-patient and out-patient services provided by the Mental Health Authority (Ministry of Health of Ghana)

^b^Data on in-patient units’ daily service users, provided by the Mental Health Authority (Ministry of Health of Ghana)

### Theme 1: The right to an adequate standard of living

**Fig. 2:**
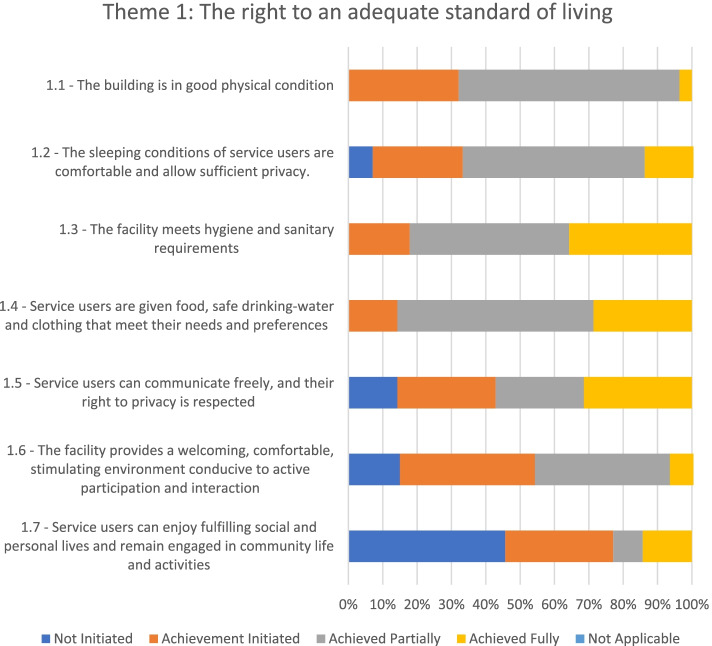
Adherence to the WHO QualityRights Toolkit’s Theme 1 in the seven mental health facilities

In regard to adherence to theme 1 (see Fig. 2), three of the mental health facilities evaluated had actively initiated changes to guarantee an adequate standard of living for service users, while the other four had partially met the standards of this theme.

Overall, most of the buildings were found suitable for use, but some of the wards were old and required structural renovations (e.g., for leaking roofs, cracks in the walls, electric wires exposed, broken windows, insufficient lighting, and absence of mosquitos’ nets). None of the mental health facilities was barrier-free for people with disabilities. In most hospitals, fire extinguishers were only available at a few vantage points, and, with some exceptions, both staff and service users were not well informed on fire safety protocols and other safety measures.

There were separate sleeping quarters for males and females in all psychiatric facilities, and the sleeping conditions of service users were generally comfortable. However, in some facilities, service users were not provided with clean mattresses and bedsheets. Furthermore, most of the sleeping wards did not have a designated place for changing clothes, and no provision was made for partitions or lockers to provide privacy to service users. Overall, washrooms and toilet facilities were found to be clean and functioning during the visits. However, bathrooms and toilets were in poor hygienic conditions in some wards and needed maintenance work (e.g., they were not cleaned regularly, toilets seats were spoilt, flushing handles were broken). The food was generally prepared under hygienic conditions, adequate, and in a sufficient quantity for the dietary needs of service users, although in some hospitals, service users had to buy their drinking water. All the facilities respected service users' right to use clothes of their choice.

Service users were allowed to receive visitors during scheduled visiting times. Generally, service users were permitted to use their own electronic gadgets (e.g., phones and laptops), although sometimes this right was restricted “depending on the service users’ condition” and “for fear such gadgets could be used to order illegal drugs into the hospital.” As a result, some service users were only allowed to use the ward phone, and their conversations were monitored by staff, so privacy was not fully respected.

The visiting committees found that the facilities’ building environment was, in general, not stimulating and conducive to interaction. Only in certain facilities, the layout of the wards promoted interaction among service users and staff, and service users could watch television or play ludo and other local board games such as oware for leisure activities.

Service users also had difficulties in remaining engaged in their communities' life and activities. In some hospitals, they were allowed to participate in activities outside the facility (but only if requested by their families).

### Theme 2: The right to enjoyment of the highest attainable standard of physical and mental health

**Fig. 3:**
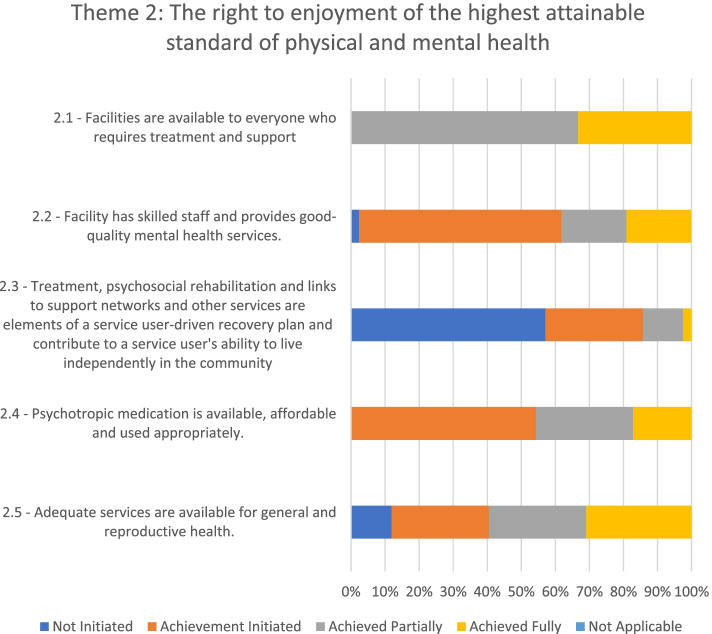
Adherence to the WHO QualityRights Toolkit’s Theme 2 in the seven mental health facilities

Adherence to theme 2 (see Fig. 3) was partially achieved in two of the psychiatric facilities evaluated and initiated in the other five.

Overall, mental health services were available, publicly funded, and accessible to the general public. The admission protocols did not discriminate based on gender, race, religion, and ethnicity. However, some of the hospitals did not admit service users with physical disabilities and children or adolescents, and no referral policy was in place. In some facilities, users or their families were asked to make a financial commitment before treatment was provided. Furthermore, service users with higher economic resources had access to special wards and could pay for a better quality of care. In all facilities, service users were found to stay sometimes longer than their planned discharge because their families were not willing to welcome them back home, and they had no economic resources for living on their own.

All the facilities had a reasonable number of psychiatric nurses, but they lacked adequate numbers of psychiatrists, psychologists, occupational therapists, and social workers. The prevalent treatment approach was medical or pharmacological, while psychosocial support, occupational therapy or rehabilitation were often unavailable due to the scarcity of professionals trained to provide them. Furthermore, staff and service users reported that the facilities sometimes experienced shortages of medications due to a lack of funding. When psychotropic medications were not available, relatives were required to procure them from pharmacies operating outside the facilities.

There was an individual treatment plan for each service user in all the facilities, although this plan was generally not comprehensive and mainly based on mental health professionals' or family members' inputs. The visiting committees found that, although service users were sometimes informed about these plans, they were seldom asked to provide their input and express their preferences on treatment and recovery.

Overall, with some exceptions, staff had limited knowledge of international and national human rights standards, such as those included in the UN CRPD and the Mental Health Act. The visiting committees also found significant gaps in staff knowledge about community services and resources to promote independent living of service users and facilitate inclusion in the community. Staff facilitated linkages mostly between service users and other community psychiatric services or the Social Welfare Department. However, the Social Welfare Department was often unable to address service users' needs due to a lack of funding.

In some of the facilities assessed, service users undergo physical health examinations and screening for physical ailments upon entry. Some hospitals also provided basic general health services, although, for specialized general health care (e.g., surgery), service users were referred to other facilities.

In most of the facilities evaluated, health education and promotion were conducted to educate service users and, in some cases, visitors. For instance, following the outbreak of the COVID-19 pandemic, the hospitals embarked on constant education on the symptoms of the infection, the mode of transmission, how it can be acquired, safety protocols, and where to receive treatment. However, there was no evidence of service users being educated on any reproductive and family planning matters.

### Theme 3: The right to exercise legal capacity and the right to personal liberty and security of person

**Fig. 4:**
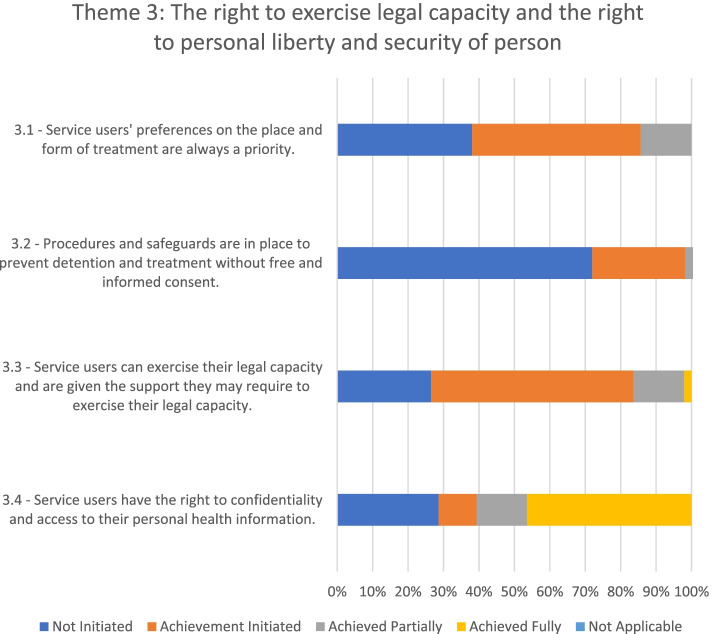
Adherence to the WHO QualityRights Toolkit’s Theme 3 in the seven mental health facilities

Six of the seven mental health facilities evaluated had taken at least initial steps toward fulfilling service users' right to legal capacity and personal liberty and security (see Fig. 4). Only one facility had not initiated changes toward fulfilling this theme.

None of the facilities prioritised service users' preferences regarding where to receive treatment or treatment options. Most of the time service users did not even know they had a right to make such decisions. Relatives usually provided informed consent regarding the type and place of treatment in consultation with the staff. Only when service users were deemed to be “stabilized” or in some instances where service users admitted themselves voluntarily into the facilities, was their informed consent sought.

Although legal avenues to appeal forced admission and treatment exist in Ghana, service users were not usually informed of this possibility. In some facilities, service users could nominate a support person to communicate their decisions, but this was not allowed in all instances. In none of the facilities were service users fully able to exercise their right to legal capacity.

Overall, all service users had personal medical files, and these were kept confidential from unauthorized access in all the facilities evaluated. However, service users had no access to their personal written information, and usually, they were not informed of their right to require access to their written records.

### Theme 4: Freedom from torture or cruel, inhuman or degrading treatment or punishment and from exploitation, violence and abuse

**Fig. 5:**
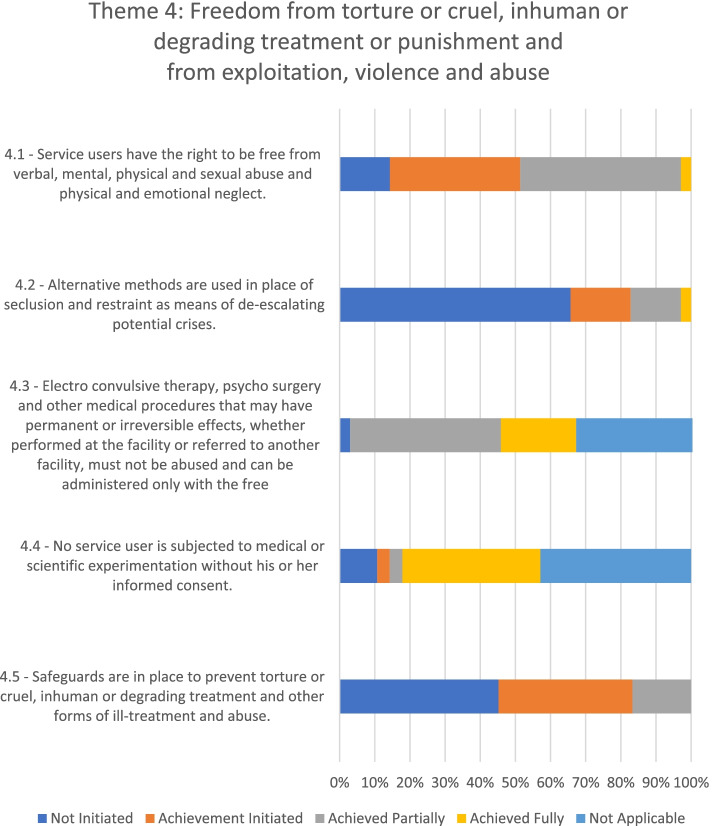
Adherence to the WHO QualityRights Toolkit’s Theme 4 in the seven mental health facilities

Five of the mental health facilities evaluated had taken initial steps toward fulfilling service users' rights to freedom from torture or cruel, inhuman, or degrading treatment or punishment and from exploitation, violence, and abuse (see Fig. 5). Only one facility had not initiated changes towards fulfilling this theme, while another facility had partially achieved this objective.

Service users told the visiting committees that they were generally treated with dignity and respect within the facilities. However, there were reports of verbal abuse by staff (e.g., yelling) and neglect was a common issue in all the facilities. There were also some reports of physical abuse as a form of punishment and control by staff. These violations were more common when staff had a heavy workload due to a lack of personnel.

Seclusion and chemical and physical restraints were found to be used in all the hospitals as a way of managing crises. The visiting committees found that sometimes these practices were also used to control “aggressive behaviors” or as a form of punishment for service users who tried to escape from the hospital or refused medication. In addition, alternative practices to seclusion or restraint (e.g., de-escalation techniques for potential crises) were rarely in place, and most of the staff had no training nor knowledge about these methods.

Not all facilities provided electroconvulsive therapy. The facilities providing electroconvulsive therapy required for the procedure either the informed consent of service users or, in line with guidelines contained in the Mental Health Act but not with the CRPD, approval of a mental health tribunal where “service users were unable to give consent”.

Medical and scientific research was conducted in some of the facilities evaluated, upon approval of the local Ethical Committee and with the informed consent of service users.

The visiting committee did not see any formal notice and information regarding the procedures for filing complaints by service users relating to abuse, violence, and neglect in the facilities evaluated. In some facilities, service users told the visiting committees that they were regularly asked verbally by nurses if they had any form of complaints and could express their concerns informally, but they were not aware of structured avenues to lodge formal complaints.

There is an independent body appointed by the government in Ghana, whose mandate is to monitor mental health facilities. However, the monitoring activities were found to be inadequate by the visiting committees.

### Theme 5: The right to live independently and be included in the community

**Fig. 6:**
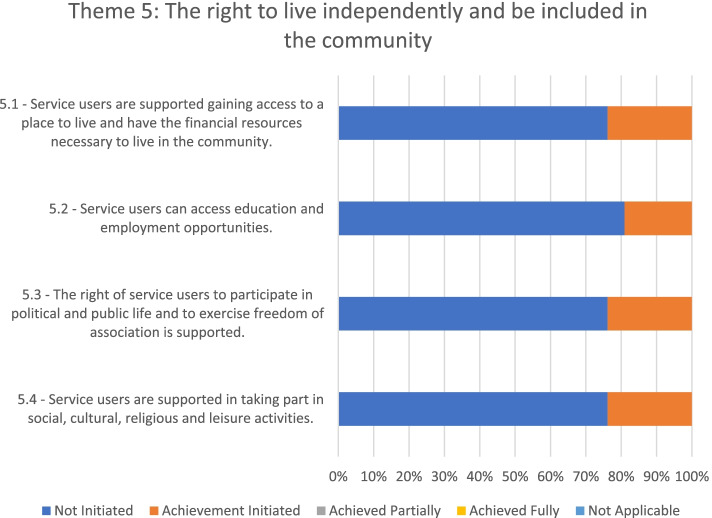
Adherence to the WHO QualityRights Toolkit’s Theme 5 in the seven mental health facilities

In regard to theme 5 (see Fig. 6), only one out of the seven mental health facilities evaluated had initiated changes toward fulfilling service users' right to live independently and be included in the community.

In Ghana, opportunities for housing and access to financial resources for service users in their communities are limited. This makes it difficult for staff to support service users in this regard. Even though there is limited funding allocated by the central government for persons with disabilities at the district level (e.g., the Livelihood Empowerment Against Poverty program), these resources are not enough to support persons with mental health conditions in education, career development, and employment opportunities. Furthermore, the mental health facilities had no resources to link service users to the services operating at the community level.

Most of the facilities evaluated did not actively support service users to participate in political life, although staff helped service users register for elections in some wards. Only in a few wards were service users provided with information on public activities they could join outside the facility upon discharge.

Table 2 shows representative verbatim quotes from the interviews.

**Table 2:** Verbatim quotes from the interviews, organized by theme

	**Representative verbatim quotes**
**Theme 1: The right to an adequate standard of living**	*“Facility is always dark when there is power outage because the generator is unable to supply power to the entire facility.” ****Service user*** *“We are not crowded because we used to be eight in a room. Also, our beds have been spread out to accommodate three beds due to the COVID-19.” ****Service user*** *“The building is not accessible to wheelchair users. The staircase is narrow and there are no lifts too. The staff or relatives or carers usually carry the physically disabled service users to the first floor of the building for treatment or admission.” ****Staff member*** *“Individual phones are prohibited for fear they could be used negatively such as arranging for weed to be brought into the facility.” ****Staff member***
**Theme 2: The right to enjoyment of the highest attainable standard of physical and mental health**	*“They make sure to look out for other underlying health conditions as well. I remember very well there was a time that they allowed for our liver to be checked. That is hepatitis B screening.” ****Service user*** *“There is no constant supply, sometimes we experience shortage of medications.” ****Staff member*** *“Yes, he did not get treatment because he could not afford.” ****Family member*** *“We are not sufficiently knowledgeable of the rights of persons with mental disabilities.” ****Staff member*** *“No staff has ever helped to come up with such comprehensive plan… we don’t have such recovery plans.” ****Service user*** *“I am only given the medicine to take, I am not told about any other thing.” ****Service user*** *“I don’t know about recovery plan.” ****Staff member*** *“Service users have no recovery plan.” ****Service user***
**Theme 3: The right to exercise legal capacity and the right to personal liberty and security of person**	*“It is not you who decides this. If you need to be admitted, you will be admitted. In this hospital, you are not the one to decide when it comes to treatment and care services.” ****Service user*** *“Consent is done on behalf of service users by their relatives often.” ****Staff member*** *“My consent was verbally sought by the staff.” ****Family member*** *“If they refuse treatment, they are forced.” ****Carer*** *“No, we don’t agree with them not to take their medication because the person may be a danger to himself and others.” ****Staff member*** *“On most occasions we don’t allow service users to refuse treatment…yes, service users have the right to refuse treatment but sometimes we force them to take the medications.” ****Staff member*** *“There are times you meet with a health official who will listen to you; at other times they don’t.” ****Service user*** *“Yes, a folder is created for each service user and information is very confidential.” ****Staff member***
**Theme 4: Freedom from torture or cruel, inhuman or degrading treatment or punishment and from exploitation, violence and abuse**	*“I was once pecked by a male staff unaware.” ****Service user*** *“Yes, a nurse held a user by the shirt and kicked him down.” ****Service user*** *“Staff members do respect us.” ****Service user*** *“Staff in this facility treat us with human rights and respect us.” ****Service user*** *“Not yet seen abuse, but I see the nurses do grumble.” ****Family member*** *“Sometime back, a service user who is a friend was physically abused as a form of punishment and control.” ****Staff member*** *“Yes, sometimes the service user may be doing something wrong, and the staff will shout at him.” ****Staff member*** *“Staff who are found abusing service users are reprimanded and service users involved are rendered an apology and counselled. Also, such incidents are recorded.” ****Staff member*** *“No standard procedure is in place, but staff are made to render an apology to the service user and queried not to behave negatively towards service users.” ****Staff member*** *“There is no avenue for complaint lodging.” ****Service user***
**Theme 5: The right to live independently and be included in the community**	*“No, we have no knowledge about the role and availability of community services or resources”. ****Family member*** *“No, we don’t have the knowledge about community services.” ****Staff member*** *“Yes, I know of department of social welfare and currently a service user is working at the laundry.” ****Service user***

## Discussion

This is the first systematic rights-based assessment of the quality of care provided to service users receiving mental healthcare in psychiatric facilities selected across several parts of Ghana. Our evaluation revealed shortcomings regarding the rights of persons with mental health conditions in psychiatric facilities in the country. The results are in consonance with those of evaluations conducted in other low- and middle-income countries [16–19] and the findings of a previous assessment carried out in Pantang Psychiatric Hospital (Ghana) [20] using the WHO QualityRights Assessment Toolkit [14].

The right to an adequate standard of living (UN CRPD, Article 28) was not fully achieved in the mental health facilities evaluated. Most of the buildings, which were all old structures, had not been renovated for a long time. Many wards needed repairs and maintenance works, but the hospitals had no financial resources to pay for them. To create an environment suitable for UN CRPD adherence, more funding should be allocated by the Ghanaian government to make the facilities disability-friendly and pay for necessary renovations. Furthermore, financial resources should be provided for installing safety equipment and the provision of training on safety measures for staff and service users.

While none of the psychiatric facilities fully achieved the right to the enjoyment of the highest attainable standard of physical and mental health (UN CRPD, Article 25), most of them had at least initiated changes toward its fulfillment. A major problem was the inconsistency of government support for medical supplies, including psychotropic medications, that, sometimes, forced hospital authorities to rely on private arrangements to obtain them. Also, family members often had to purchase medications for service users in pharmacies outside the facilities. The document review and the interviews also revealed that the hospitals did not have sufficient mental health professionals to meet the needs of service users seeking care. Although psychiatric nurses were present in adequate numbers, there was a lack of psychiatrists, psychologists, occupational therapists, and social workers, amongst the others. As a result, service users had little or no access to non-pharmacological interventions. Further efforts to train and hire mental health providers with different skills, able to provide a diverse range of interventions, should be made by the government and hospital authorities. In line with numerous studies in different countries [23–25], mental health workers in Ghana were found to have limited knowledge of international human rights standards. To address this problem, the Mental Health Authority is currently providing face-to-face and online QualityRights training on human rights in mental health in some of the facilities assessed [26].

The visiting committees found serious shortcomings in the adherence to the CRPD requirement for service users to have a right to exercise legal capacity (UN CRPD, Article 12) and to personal liberty and security (UN CRPD, Article 14). Service users were usually unaware they had a right to make decisions about their lives, including decisions about their treatment and place of care. Family members generally provided informed consent in consultation with staff, and the input of service users was rarely sought. These findings align with data from previous studies carried out in Ghana that show a strong endorsement of paternalistic and socially restrictive attitudes towards people with mental health conditions [27]. For instance, a recent study conducted in Ghana found that more than 40% of the respondents saw no problem in denying persons with mental health conditions their rights, while 68.3% believed that they required “the same kind of control as children” [27]. To promote service users’ rights to legal capacity and personal liberty and security, it would be fundamental to educate staff, service users, and carers to ensure that informed consent of service users is sought before admission, treatment, and in all decisions regarding service users’ lives. Service users should also be provided with information, in accessible formats, about their rights and the legal opportunities and appeal procedures available to redress potential violations. Staff, service users, and carers could benefit from training on working together to develop recovery plans and advance directives.

There were reports of violations of the rights to freedom from torture or cruel, inhuman, or degrading treatment or punishment and from exploitation, violence, and abuse (UN CRPD, Articles 15 and 16) in all the facilities evaluated. The visiting committees observed instances of verbal and physical abuse against service users. Neglect was also a significant issue in all the facilities. Seclusion and restraints were often used as a way of managing crises. However, the visiting committees found that sometimes seclusion and restraint were also used to control “aggressive behaviors” or as a form of punishment, although the Mental Health Act forbids this. In addition, alternative practices to the use of coercion were seldom in place, and most of the staff had no training nor knowledge about these methods. The UN CRPD demand States to end the use of seclusion and restraint and replace these with practices that align with people’s will and preferences. Although this objective will require time to be achieved, it is essential that facilities and staff in Ghana make more efforts to implement alternatives and put in place measures to avoid the use of involuntary practices in the future. Training for staff and service users on de-escalation techniques for potential crises, advance directives, and supported-decision making could help achieve this objective. It could also be useful to develop action plans setting out goals and deadlines for implementing alternatives to the use of seclusion and restraint in each facility.

Our assessment also revealed that most mental health facilities evaluated had not initiated changes toward fulfilling service users' right to live independently and be included in the community. This was mainly due to a general lack of formal opportunities in Ghana for housing, education, or employment for people with mental health conditions. However, Ghana has numerous civil society organizations and organizations of people with disabilities working in the mental health field (e.g., Mental Health Society of Ghana, MindFreedom Ghana, BasicNeeds Ghana, Special Olympics Ghana) and advocating for service users’ rights. These organizations partner with the Mental Health Authority to create more opportunities at the community level for people with mental health conditions and promote their right to living independently.

Based on the results of our evaluation, some critical steps to improve the quality of care and respect of human rights in mental health facilities are underway in Ghana. In collaboration with WHO, the Mental Health Authority and local organizations of persons with mental health conditions are working together with hospital authorities, staff, and service users to develop improvement plans in the facilities assessed.

Our study presents several strengths: The high number of interviews conducted in each facility; the rigorously trained visiting committees; the use of a structured and comprehensive evaluation instrument, the WHO QualityRights Assessment Toolkit; the inclusion of people with mental health conditions and their organizations in all phases of the project; and the inclusion of mental health facilities located in different Ghanaian regions that allowed us to obtain a representative picture of the quality of care and respect of human rights in the mental health system in Ghana. However, the study also presents some limitations that are worth highlighting. For instance, different visiting committee members were employed in the visits to the different facilities. However, a core group of four visiting committee members was present during all the visits and participated in all discussions on the scoring and reporting to reduce subjectivity and increase reliability of the findings. Furthermore, all the visits were scheduled, so we cannot exclude that the hospital authorities prepared for the visit by “cleaning up” the facility so that committee members will see a “sanitized” version of it rather than the actual conditions, a situation that might indicate that some of our findings represent conservative evaluations of the true situation. However, this limitation was addressed by asking our interviewees (service users and staff) to provide information using a typical day as their reference.

## Conclusions

The data from our study and the recommendations provided may be a valuable resource for Ghanaian stakeholders to develop improvement plans and implement a human rights-based approach in mental health facilities throughout the country. Furthermore, the strong commitment of the Mental Health Authority to mental health service reform as well as the collaboration of the Ghanaian organizations working on the ground, and of hospital management in the facilities assessed for this study provides encouraging signs that improvement along the lines suggested in this paper may be forthcoming.

## Data Availability

The datasets used and analysed during the current study are available from the corresponding author on reasonable request.

## Abbreviations

UN CRPD: United Nations Convention on the Rights of Persons with Disabilities
WHO: World Health Organization
